# Cytosolic calcium ions exert a major influence on the firing rate and maintenance of pacemaker activity in guinea-pig sinus node

**DOI:** 10.3389/fphys.2015.00023

**Published:** 2015-02-10

**Authors:** Rebecca A. Capel, Derek A. Terrar

**Affiliations:** Department of Pharmacology, University of OxfordOxford, UK

**Keywords:** sino-atrial node, heart rate, pacemaking, cytosolic calcium, calcium chelation

## Abstract

The sino-atrial node (SAN) provides the electrical stimulus to initiate every heart beat. Cellular processes underlying this activity have been debated extensively, especially with regards to the role of intracellular calcium. We have used whole-cell application of 1,2-bis(o-aminophenoxy)ethane-N,N,N′,N′-tetraacetic acid (BAPTA), a rapid calcium chelator, to guinea pig isolated SAN myocytes to assess the effect of rapid reduction of intracellular calcium on SAN cell electrical activity. High-dose (10 mM) BAPTA induced rapid and complete cessation of rhythmic action potential (AP) firing (time to cessation 5.5 ± 1.7 s). Over a range of concentrations, BAPTA induced slowing of action potential firing and disruption of rhythmic activity, which was dose-dependent in its time of onset. Exposure to BAPTA was associated with stereotyped action potential changes similar to those previously reported in the presence of ryanodine, namely depolarization of the most negative diastolic potential, prolongation of action potentials and a reduction in action potential amplitude. These experiments are consistent with the view that cytosolic calcium is essential to the maintenance of rhythmic pacemaker activity.

## Introduction

It has been proposed that cytosololic calcium including that released from the sarcoplasmic reticulum (SR) plays an important role in the generation of pacemaker activity in both mammalian and amphibian pacemaker tissue (Rigg and Terrar, 1996; Ju and Allen, 1998, 1999; Rigg et al., 2000), as well as in subsidiary pacemaker (Zhou and Lipsius, 1993) and atrioventricular cells (Hancox et al., 1994). It has been suggested that uptake and release of calcium by the SR could provide a timing mechanism for pacemaking that is referred to as the “calcium clock” (Vinogradova et al., 2004), and in recent years there has been vigorous debate concerning the relative importance of such a “calcium clock” and the more conventional “membrane clock” dependent on activation and de-activation of membrane ion channels (Lakatta and Difrancesco, 2009; DiFrancesco and Noble, 2012; Maltsev and Lakatta, 2012).

An important challenge to the possible importance of cytosolic calcium for pacemaking was provided by the work of Himeno et al. (2011) who recorded spontaneous electrical activity in guinea pig isolated pacemaker myocytes under perforated patch conditions, and then ruptured the membrane beneath the patch to apply the calcium chelator BAPTA to the cytosol from the patch pipette solution. Under these conditions, spontaneous action potentials were observed to continue at least for 30 s in the presence of cytosolic BAPTA, although pacemaker activity did become erratic or stop after several minutes. The observations were thought not to be consistent with a major role for cytosolic calcium in controlling pacemaker activity, at least for short term (ms or seconds) mechanisms. These observations have in turn been challenged, at least in part on the basis of arguments concerning possible changes in the seal resistance (Maltsev et al., 2011; Yaniv et al., 2013).

The aim of the experiments presented here was to further examine this question in guinea pig pacemaker myocytes isolated from sino-atrial node. We have used techniques similar to those of Himeno et al. (2011), as well as conventional ruptured patch approaches with several concentrations of BAPTA applied from the patch pipette.

## Materials and methods

Guinea pig sino-atrial node myocytes were isolated as described previously (Rigg et al., 2000). Briefly, guinea-pigs were killed by concussion followed by cervical dislocation, the heart rapidly removed, placed into heparin-containing zero-calcium modified Tyrode solution (in mM: NaCl 136, KCl 5.4, NaHCO3 12, Na^+^ pyruvate 1, NaH_2_PO_4_ 1, MgCl_2_ 1, glucose 5, ethylene glycol tetraacetic acid (EGTA) 0.04; gassed with 95% O_2_/5% CO_2_ to maintain a pH of 7.4) and then mounted on a Langendorff apparatus for retrograde perfusion (zero-calcium modified Tyrode without addition of EGTA). The heart was enzymatically digested (Worthington Type II Collagenase, Worthington Biochemical Corp), atria removed and the SAN dissected into small strips under a microscope. Single cells were isolated by trituration in warmed, oxygenated high-potassium storage solution (in mM: KCl 70, MgCl_2_ 5, K^+^-glutamine 5, taurine 20, EGTA 0.04, succinic acid 5, KH_2_PO_4_ 20, HEPES 5, glucose 10; pH to 7.2 with KOH) and then transferred directly to 4°C for storage in the same solution until use.

Standard whole-cell patch solution contained (in mM): K^+^-aspartate 110, KCl 10, NaCl 5, MgCl_2_ 5.2, HEPES 5, K_2_ATP 5, pH to 7.2 with KOH. Amphotericin was dissolved in dimethyl sulphoxide (DMSO) to form a stock solution (20 mg ml^−1^) and then diluted into patch solution to achieve a final concentration of 240 μg ml^−1^. 1,2-Bis(2-aminophenoxy)ethane-N,N,N′,N′-tetraacetic acid tetrapotassium salt (BAPTA) was dissolved in double-distilled water and diluted into whole-cell patch solution as appropriate.

During experiments, cells were superfused with physiological saline solution (PSS) at 35 ± 2°C (in mM: NaCl 125, NaHCO_3_ 25, KCl 5.4, NaH_2_PO_4_ 1.2, MgCl_2_ 1, glucose 5.5, CaCl_2_ 1.8, pH to 7.4 with NaOH and oxygenated with 95% O_2_/5% CO_2_). Within a given experiment, temperature fluctuation was <0.5°C.

**Figure 1:**
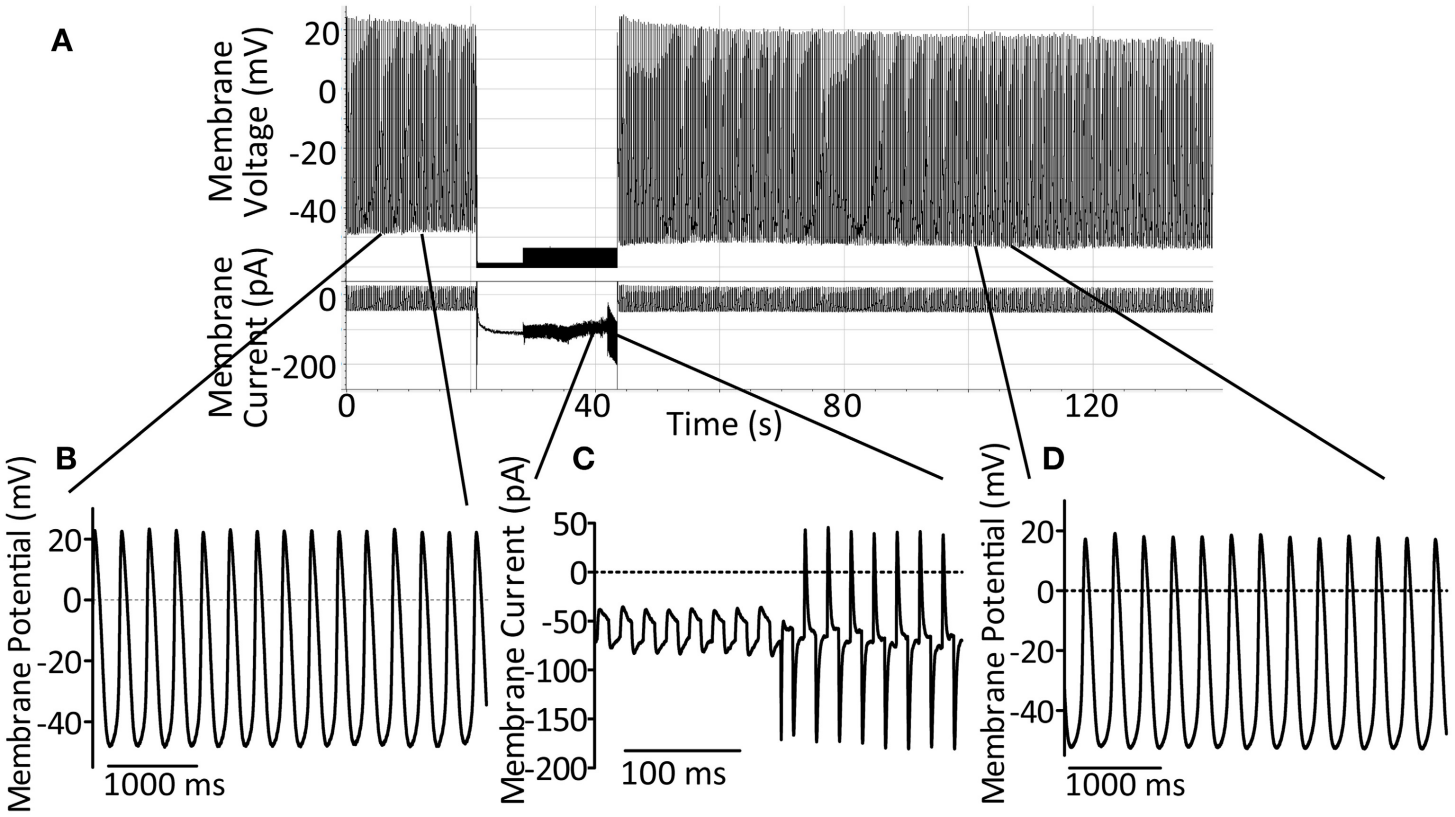
**(A)** Demonstration of the perforated-to-whole-cell patch method with control patch solution. After formation of a GΩ seal, up to 15 min is allowed for perforation of the membrane (amphorericin B, 240 μg ml^−1^). Action potentials are recorded in the perforated patch configuration for at least 10 s before switch to voltage clamp at −60 mV using an AxoPatch200B amplifier. Seal integrity can then be monitored and recorded using the amplifier-controlled seal test. Whole-cell access is achieved by rapid suction and confirmed by the onset of large capacitative transients. After gaining whole-cell access the amplifier is rapidly switched back to current clamp mode in order to follow spontaneous action potential firing once again. **(B)** An expanded section of trace, as indicated, to demonstrate control action potentials in the perforated patch configuration. **(C)** An expanded section of trace, as indicated, to demonstrate the seal test signal during patch rupture to achieve whole-cell access. **(D)** An expanded section of trace, as indicated, to demonstrate the maintenance of spontaneous action potential firing and expected action potential shape 60 s after patch rupture under control conditions.

For perforated patch recording, micropipettes of 3–6 MΩ were manufactured from borosilicate glass (GC100F, Harvard Apparatus) using a two-step gravity-driven puller (PP-83, Narishige, Japan). Pipettes were mounted on a CV203BU headstage and recordings made using an AxoPatch200B amplifier with pClamp7 software. GΩ seals were formed using manual suction and up to 15 min allowed for stable perforation. Action potentials were recorded in current clamp mode, cells were switched to voltage clamp in order to monitor seal integrity and whole-cell access achieved by manual suction. A holding potential of −60 mV was used during this process. Upon whole-cell access, judged by appearance of capacitative transients, the amplifier was rapidly switched back to current clamp mode and cellular action potentials were monitored until cessation of rhythmic activity. A representative control trace to demonstrate this method is presented in Figure 1A. A representative section of action potentials recorded during perforated patch, the transition from perforated to whole-cell patch and a representative section of action potentials in whole-cell configuration are shown expanded in Figures 1B–D respectively.

During experiments in which only whole-cell recordings were carried out, the same method was followed without the addition of amphotericin to the whole-cell patch solution. A holding voltage of −40 mV was used during confirmation of whole-cell access in these experiments since this was found to minimize damage to the seal during rupture of the patch. Where Fluo-5F was used for illustration of rhythmic firing before patch rupture this was applied as 3 μM of the cell-permeant form Fluo-5F-AM (Invitrogen, UK) by incubation at room temperature for 10 min followed by a further 10 min of superfusion with PSS to allow de-esterification.

## Results

### Intracellular calcium is a requirement for pacemaking activity

Our first aim was to repeat the experiments of Himeno et al. (2011). We reasoned that, if cytosolic calcium has no effect on cellular beating rate, then carrying out experiments in the absence of amphotericin would allow us to confirm that the perforation technique itself was not causing any confounding effects. Under these conditions the addition of 10 mM BAPTA to the patch pipette led to rapid cessation of rhythmic cellular activity with an average time to cessation of 5.5 ± 1.7 s from patch rupture (*n* = 6). In contrast, although control cells showed a gentle rate decline (9 ± 4% reduction after 60 s, *n* = 3) which was statistically-significant at 90 s post access (20 ± 3% reduction, *n* = 3), cells exposed to our standard whole-cell patch solution maintained rhythmic activity for over 5 min.

**Figure 2:**
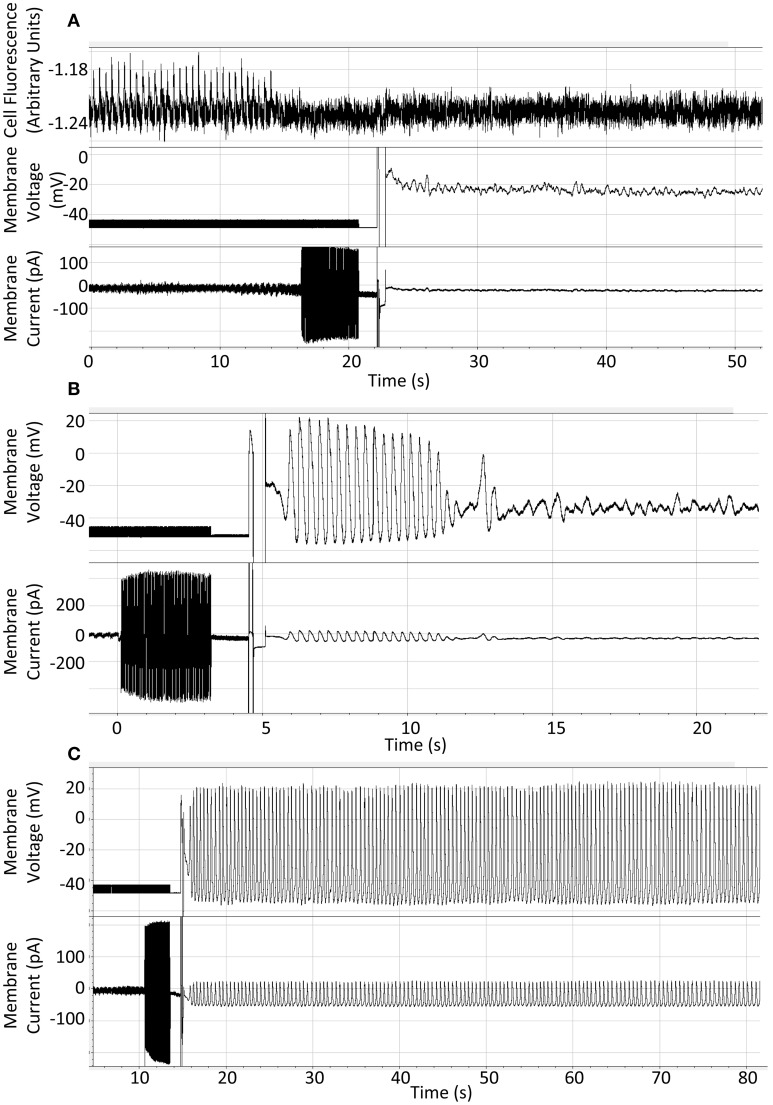
**(A)** Representative recording to show result of patch-application of 10 mM BAPTA to an isolated guinea pig SAN cell with fluo5F included to demonstrate rhythmic activity under control conditions. **(B)** Representative recording to show rapid cessation of spontaneous activity on application of 10 mM BAPTA from the patch pipette solution. **(C)** Representative recording to show continuation of expected spontaneous rhythmic action potential generation when whole-cell access is gained using standard whole-cell patch solution. Cells were superfused with Physiological Saline Solution at 35 ± 2°C throughout.

We performed some of these experiments after loading cells with the calcium indicator Fluo5F to demonstrate that rhythmic cellular activity was indeed present before rapid chelation of calcium, as it was common that cells stopped before the switch to current clamp could be completed. There was no difference in the response to BAPTA under these conditions. A representative trace of 10 mM BAPTA exposure with preceding calcium signal is presented in Figure 2A. The response of a cell to 10 mM BAPTA in the absence of Fluo5F, which maintained action potentials for several seconds after patch rupture, is presented in Figure 2B and can be compared to that of a control cell shown in Figure 2C.

### Effect of intracellular calcium chelation on action potential waveforms

The rapid cessation of action potential firing witnessed in our first set of recordings did not allow us to compare action potential waveforms seen over time. We therefore reverted to a direct repetition of the Himeno et al. (2011), experiments, recording control action potentials by perforated patch before BAPTA application by patch rupture and whole-cell access.

**Figure 3:**
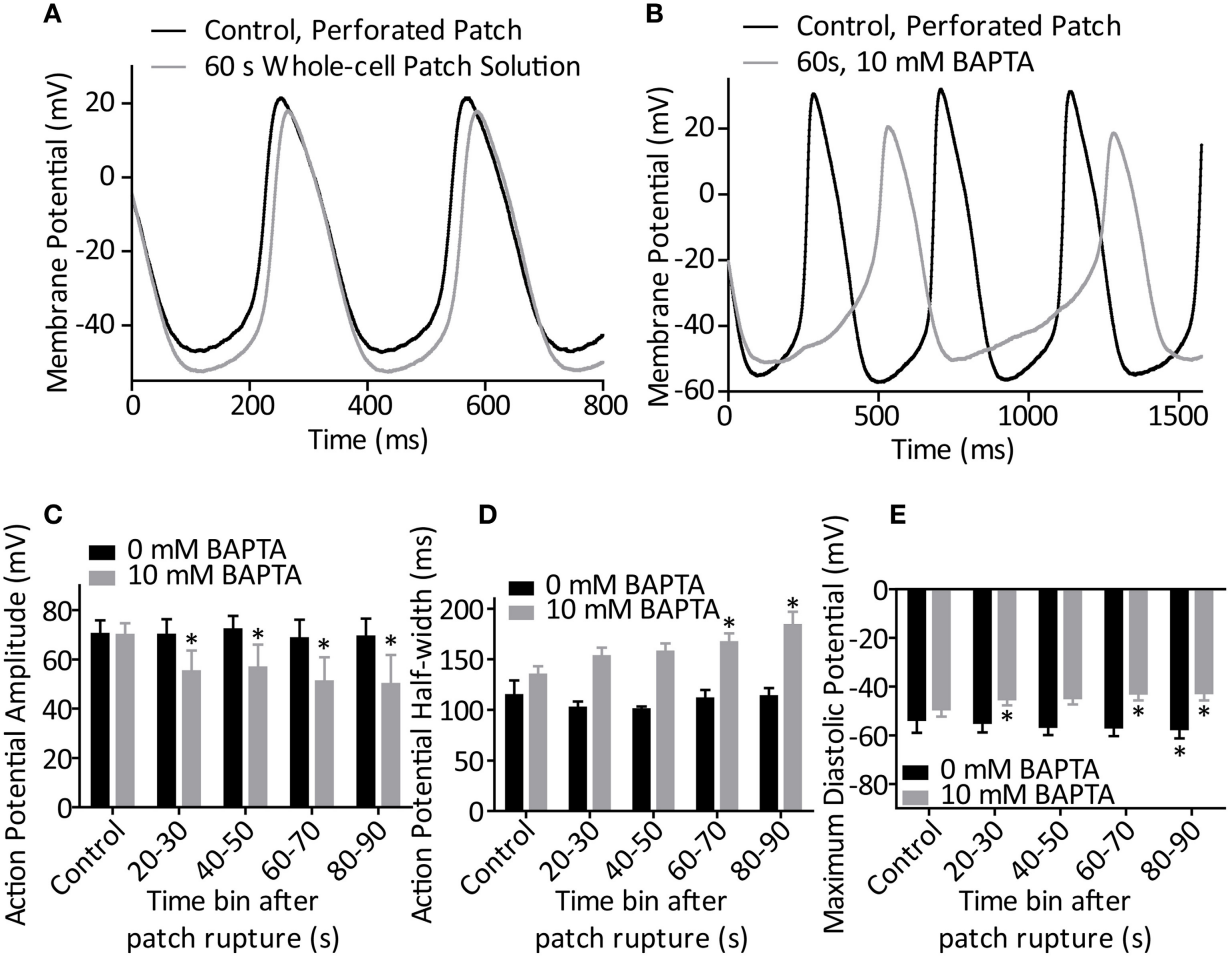
**(A)** Representative action potentials recorded during perforated patch control and 60 s after patch rupture with standard whole-cell patch solution (0 mM BAPTA). **(B)** Representative action potentials recorded during perforated patch control and 60 s after patch rupture to apply 10 mM BAPTA. **(C)** Effect of patch rupture on action potential amplitude over the course of 90 s. 10 mM BAPTA significantly reduced AP amplitude (*p* < 0.05, One-Way ANOVA with repeated measures). **(D)** Effect of patch rupture on half-width of the action potential. 10 mM BAPTA significantly lengthened the action potential half-width (*p* < 0.05, One-Way ANOVA with repeated measures). **(E)** Effect of patch rupture on most negative diastolic potential over the course of 90 s. 10 mM BAPTA significantly depolarized the MDP (*p* < 0.05, One-Way ANOVA with repeated measures) ^*^Denotes significant difference from control, during perforated patch recording (*p* < 0.05 by *post-hoc* test with Dunnett's multiple comparison performed after One-Way ANOVA). *n* = 4 for 0 mM and *n* = 5 for 10 mM recordings.

After exposure to amphotericin/DMSO by whole-cell access, SAN cell appearance became markedly changed over the course of 5 min, exhibiting cell swelling or membrane bulging. To minimize any confounding effects of these phenomena we assessed cellular rate over the first 90 s post whole-cell break-in only and then followed activity until perturbation of rhythmic action potential firing. Action potentials fired in each 10 s timebin from patch rupture were analyzed for morphology regardless of whether cell firing at the time was rhythmic or sporadic. Representative traces of the 0 and 10 mM BAPTA conditions presented in Figures 3A,B.

During BAPTA-induced cell slowing (*n* = 5) several changes were observed in action potential waveforms. BAPTA induced a significant reduction in action potential amplitude over time (*p* < 0.05, One-Way repeated measures ANOVA, Figure 3C), and prolongation of action potential half-width (*p* < 0.05, One-Way ANOVA, Figure 3D) whilst these values were unchanged in control cells (both *p* > 0.05, One-Way ANOVA with repeated measures, *n* = 4). Further, whilst exposure of cells to our standard whole-cell patch solution led to a significant hyperpolarization of the most negative diastolic potential over time (*p* < 0.05, One-Way ANOVA, *n* = 4), inclusion of 10 mM BAPTA in the patch solution led to a significant depolarization of this measure (*p* < 0.05, One-Way ANOVA, Figure 3E). No change was seen in the maximum rate of action potential upstroke under either condition (both, *p* > 0.05, separate One-Way ANOVA analyses, data not shown).

### Cellular firing is dose-dependently affected by intracellular calcium chelation

During control recordings using our standard intracellular patch solution, conversion of cells from perforated patch to whole-cell patch led to in initial increase in rate followed by gentle slowing (*p* < 0.05, One-Way repeated measures ANOVA, *n* = 4). *Post-hoc* testing with Tukey correction revealed that action potential firing only became significantly less frequent than that during the perforated patch at 90 s after patch rupture.

**Figure 4:**
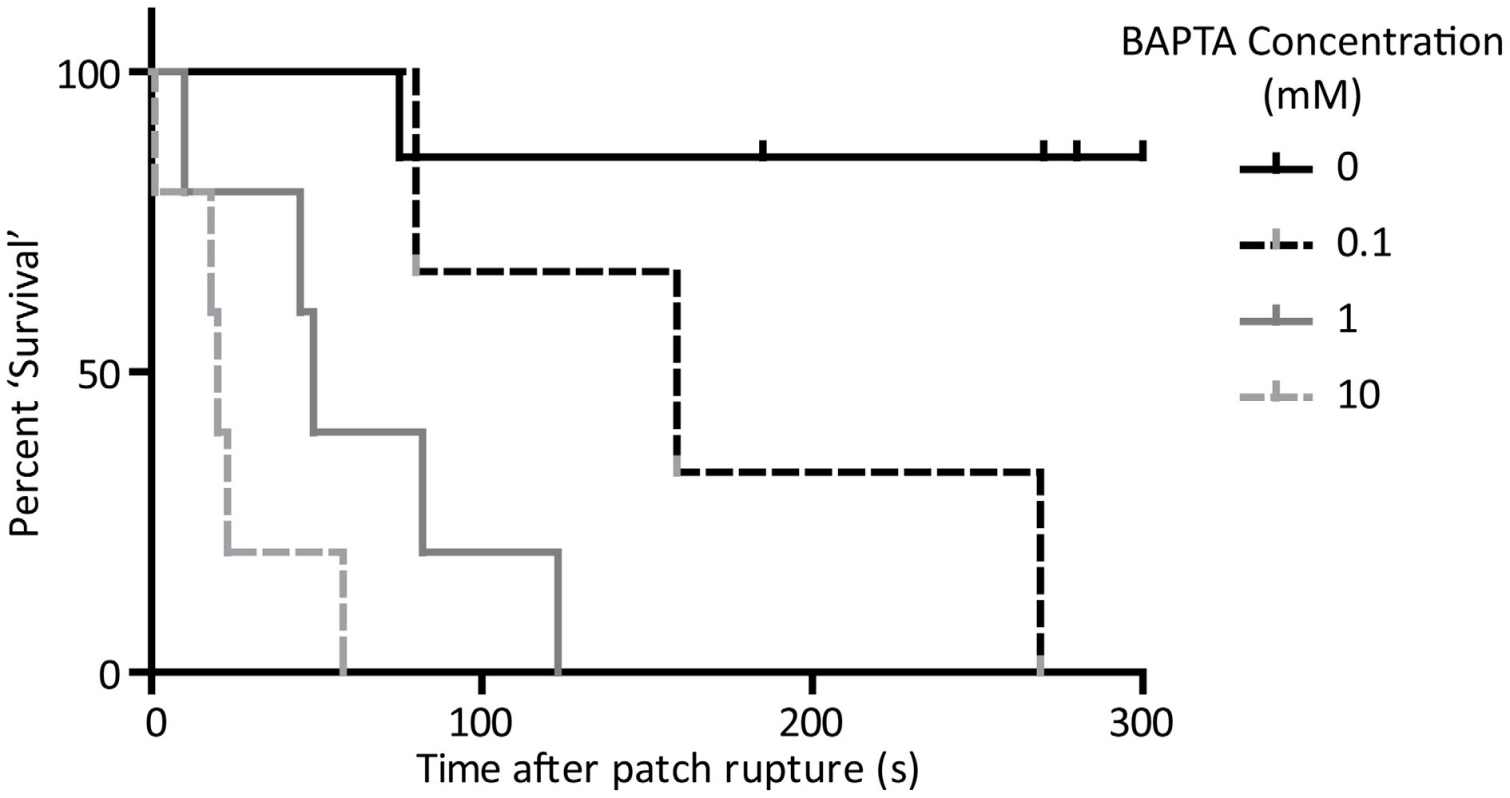
**“Survival” curve to show cessation of rhythmic activity on application of BAPTA by the perforated-to-whole-cell patch method**. Time taken for cells to miss the firing of an expected action potential was significantly associated with the concentration of BAPTA included in the patch solution (*p* < 0.05, Log-rank comparison of survival curves). *n* = 5 for 0 mM, 3 for 0.1 mM, 5 for 1 mM, and 5 for 10 mM conditions.

We next carried out action potential recordings by the perforated-to-whole-cell patch method at a range of BAPTA concentrations. Whole-cell application of BAPTA led to a dose-dependent perturbation of rhythmic activity. The time taken for a given cell to miss the expected firing of an action potential was longer than 3 min in all but one control cell (which missed one AP at 75 s post patch rupture and then returned to rhythmic firing and continued for longer than 3 min). In the presence of 0.1 mM BAPTA average time to miss a beat was 169 ± 55 s (*n* = 3). For the 1 mM condition, this shortened to 62 ± 19 s (*n* = 5) and for the 10 mM condition to 24 ± 9 s (*n* = 5). A “survival” plot of time to first pause in activity is presented in Figure 4. There is a significant effect of BAPTA concentration (*P* < 0.0001) as assessed by Log-Rank comparison of survival curves.

**Figure 5:**
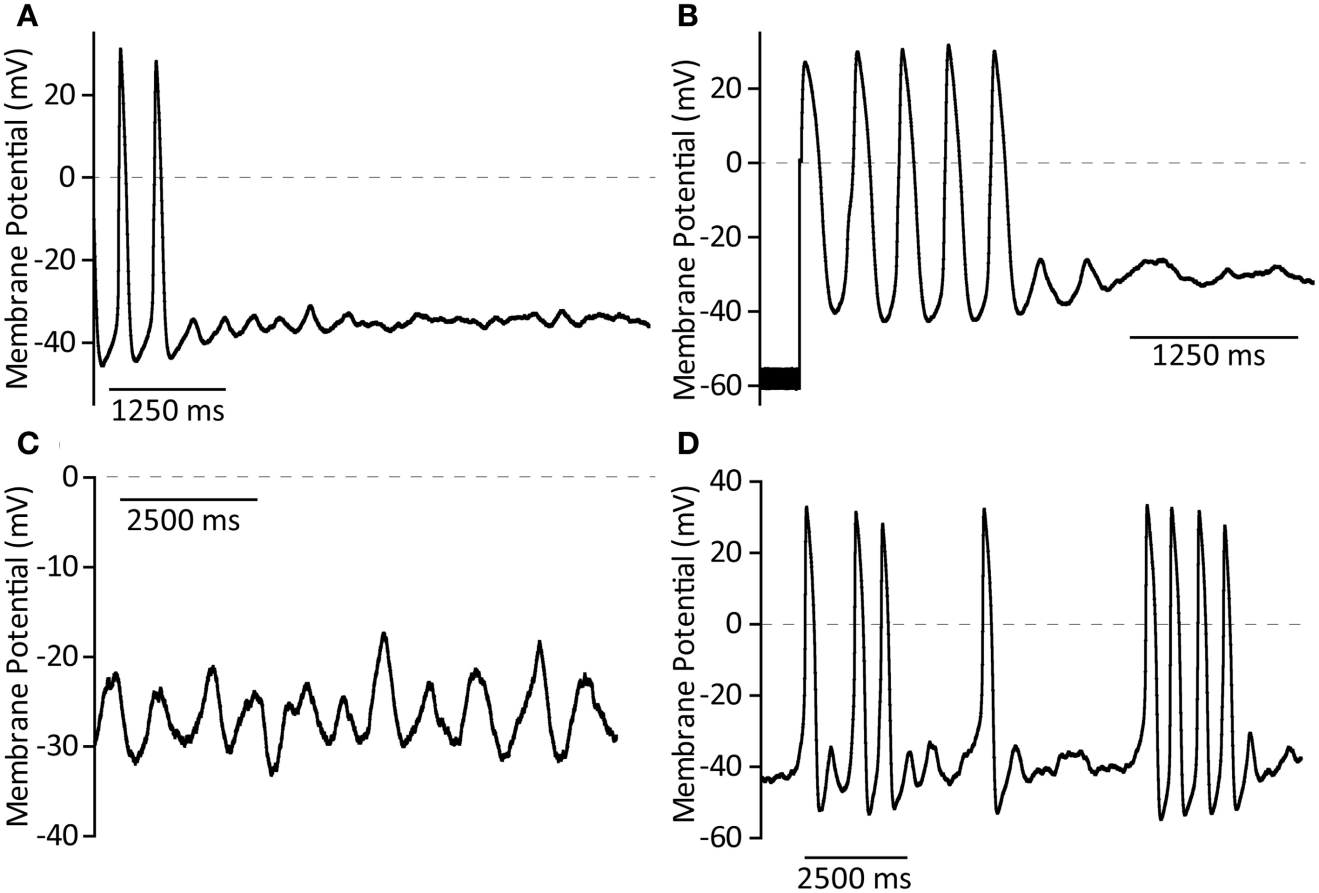
**Representative examples of membrane potential phenomena after cessation of true cell rhythmicity caused by exposure to BAPTA in the patch solution. (A)** Complete cessation of action potential firing and quiescence around −35 mV. **(B)** Stimulation of a short train of action potentials by anode break excitation followed by return to quiescence. **(C)** Rhythmic membrane potential fluctuations which fail to convert to full action potential firing. **(D)** Sporadic burst firing activity.

Data are presented as time taken to miss an action potential because cellular behavior observed after this point was variable. In all cases cells ceased true rhythmic activity after missing one or more APs. Some cells rapidly fell into complete cessation, with membrane potential fluctuating in the region of −30 to −40 mV (See Figure 5A). In these cells, regardless of BAPTA concentration, application of hyperpolarizing voltage clamp to −60 mV and subsequent relief was able to induce firing of one or more action potentials by anode-break excitation (rebound excitation seen after injection of hyperpolarizing current), demonstrating that membrane currents associated with normal activity remained functional (Figure 5B). During quiescent periods it was common for cells to achieve a rhythmic fluctuation in cellular membrane potential which did not induce full action potential firing (Figure 5C). Further cells were seen to fire single action potentials at random or else commenced burst-like activity in which short trains of 3–5 action potentials could be observed occurring with little predictability (Figure 5D). Cells were not necessarily limited to a single one of these behaviors.

Due to the propensity of cells to continue sporadic firing after cessation of “normal” activity, or to exhibit membrane potential fluctuations which did not stimulate a full action potential, analysis of rate using our usual method (determination of dominant firing frequency by spectral analysis) was not deemed accurate for this purpose. Instead, we assessed the number of full action potentials fired over each 10 s period from patch rupture, where an action potential was defined as a spontaneously-fired event which overshot −10 mV or reached 50% of control action potential amplitude. As with data presented in Figure 3, action potentials included in this analysis were those considered to have fired regardless of rhythmic cell behavior or otherwise.

**Figure 6:**
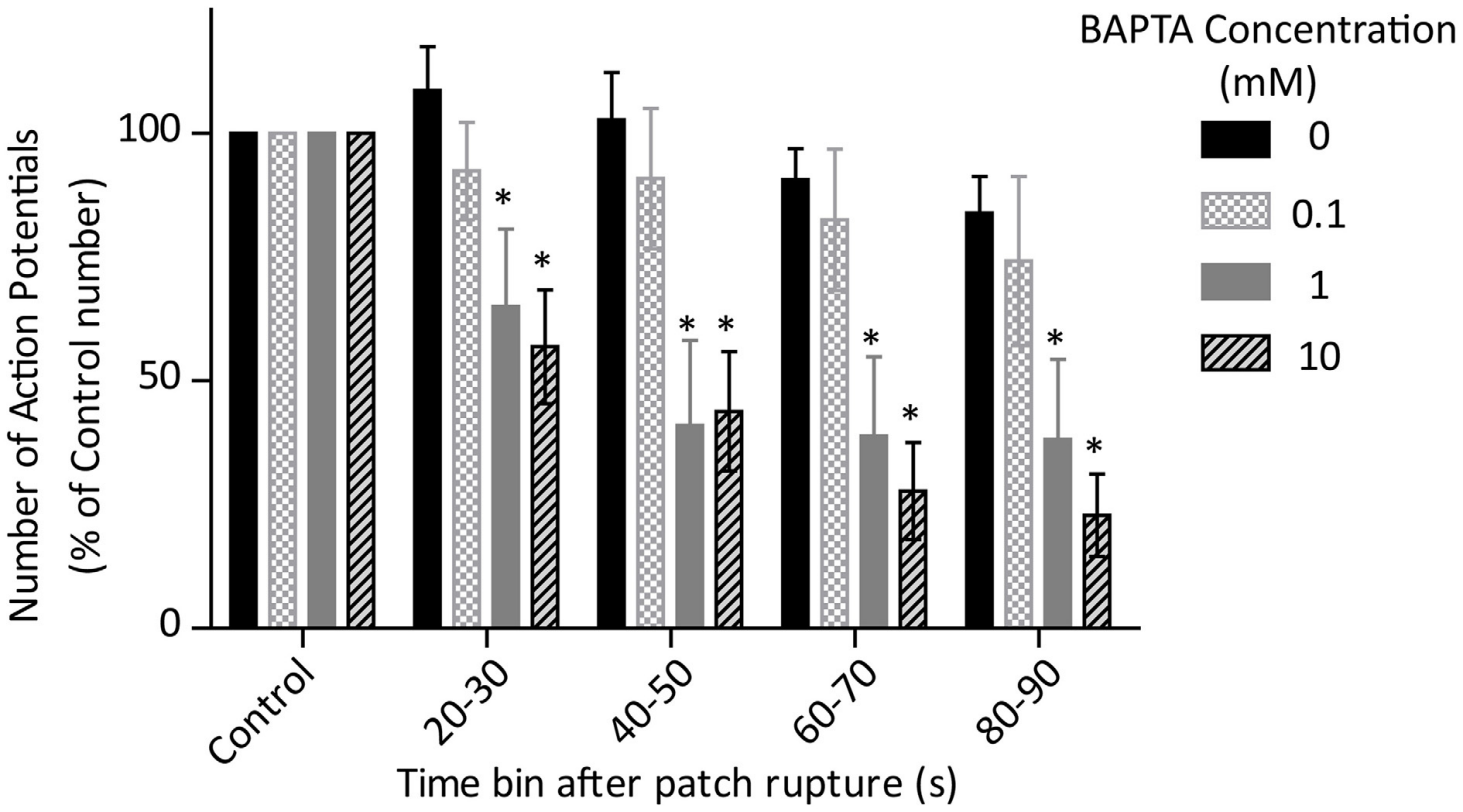
**Effects of control (0 mM, *n* = 4) and 0.1 (*n* = 3), 1 (*n* = 5) and 10 (*n* = 5) mM BAPTA on cell firing rate, measured as the number of action potentials fired during each 10 s time bin after rupture of the cell membrane and conversion of perforated patch to whole-cell access**. There is a significant effect of BAPTA, time and also an interaction (all *p* < 0.05 by Two-Way ANOVA). ^*^Denotes significant difference (*p* < 0.05) from 0 mM by *post-hoc* comparison with Bonferoni correction

The effect of BAPTA on the number of fired action potentials over time is presented in Figure 6. Two-Way ANOVA analysis showed a significant effect of BAPTA concentration and of exposure time on cell firing frequency (both *p* < 0.05). However, there was also an interaction effect which suggests that the timecourse of BAPTA-mediated slowing differs between concentrations (*p* < 0.05). *Post-hoc* testing with Bonferroni corrections reveals a significant effect of both the 1 and 10 mM BAPTA concentrations in comparison to control, but no difference between these two concentrations over all time bins.

### Confounding effects of patch method on results of BAPTA application

**Figure 7:**
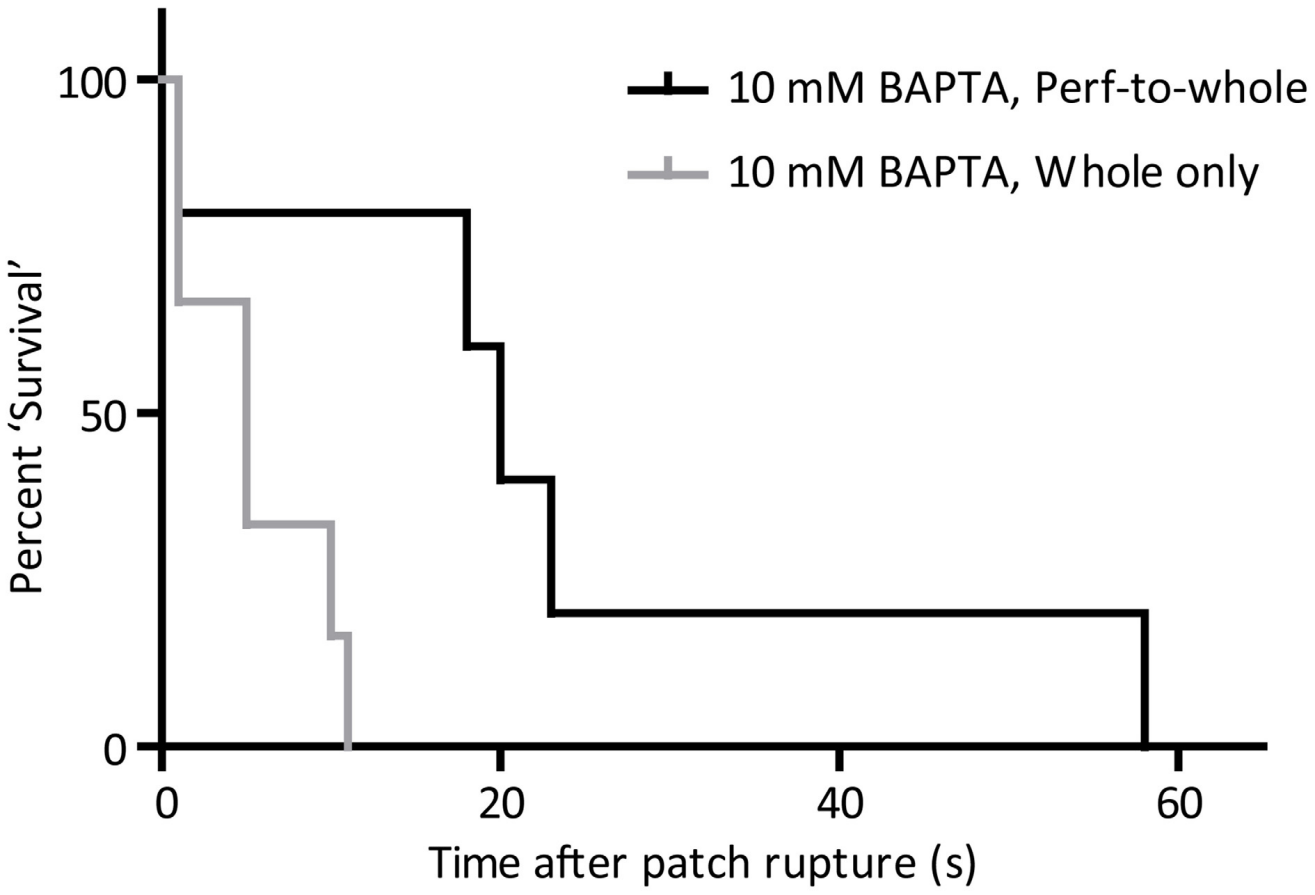
**“Survival” curve to show the difference in effect of 10 mM BAPTA when applied from the perforated patch (*n* = 5) and whole-cell (*n* = 6) only configurations**. Time taken for cells to miss the firing of an expected action potential was significantly longer when BAPTA was applied in the whole-cell patch solution during the perforated patch method (*p* < 0.05, Log-rank comparison of survival curves).

Direct comparison of the effect of 10 mM BAPTA on cell firing by the whole-cell only and the perforated-to-whole-cell patch method show that cell “survival” is much more pronounced in the presence of the ionophore and its solvent (*p* < 0.05, Log-Rank test of “survival” time to first missed beat, Figure 7). Cells exposed to 10 mM BAPTA alone succumbed to the effects of chelation in 5.5 ± 1.7 s and were most likely to reach a quiescent or sub-threshold firing state. When amphotericin and DMSO were also included in the patch solution, perturbation of rhythm was seen at 24 ± 9 s and cells demonstrated the full range of behaviors described above.

## Discussion

The data presented in this paper are consistent with the proposal that the presence of intracellular calcium is an essential condition for the maintenance of rhythmic action potential firing in guinea pig sino-atrial node cells. All cells exposed to BAPTA, at a range of concentrations, experienced a derangement of rhythmic action potential firing which was not seen under control conditions. The time taken for cells to miss firing an expected action potential was dose-dependently related to BAPTA exposure. Although there is a time-dependent effect of BAPTA applied via the patch pipette, it would seem that there is not a distinct dose-response curve when the number of action potentials fired during each 10 s time bin is considered. The interpretation of these rate data is not straightforward since a range of cell firing characteristics was seen on cessation of true rhythmic activity. Taken together, these observations may suggest the gradual reduction of cytosolic calcium to a threshold level at which the cell no longer supports rhythmic activity as opposed to an effect of different chelation levels on different signaling pathways.

Rapid chelation of intracellular calcium during our whole-cell only experiments often resulted in cessation of rhythmic firing activity before the amplifier could be switched away from the seal-test mode and into current clamp. Previous work from this laboratory has shown that rapid switch (<1 s transition) of guinea pig SAN cells into BAPTA-AM leads to very rapid cessation of action potential firing. There is evidence that BAPTA-AM is capable of blocking voltage-gated potassium channels (Tang et al., 2007) which is the major reason why the effect of BAPTA application by whole-cell access is important to test. The data presented in this paper are therefore in agreement with previous studies which have used membrane-permeant chelators to investigate cell dependence on cytosolic calcium (Vinogradova et al., 2000; Sanders et al., 2006).

The action potential shape after exposure to BAPTA is distinctly different from those seen in control. In the absence of calcium directly beneath the membrane it would be expected that L-type calcium channels open for longer due to a reduced stimulus for calcium-dependent inactivation (Himeno et al., 2011). In this regard, our data are in agreement with the observations of Noma's group (Himeno et al., 2011) in exhibiting significant action potential prolongation. Cytosolic calcium also enhances delayed rectifier potassium currents, particularly I_Ks_ (Xie et al., 2015). Cytosolic calcium chelation would therefore be expected to reduce the repolarizing potassium current and both prolong action potential duration and lead to depolarization of the most negative diastolic potential, which is indeed seen in our cells.

Chelation of intracellular calcium is also likely to inhibit activation of the funny current I*_f_* (Rigg et al., 2003) and lead to slowing of rhythmic AP firing. Sino-atrial node cells are thought to maintain a diastolic cAMP (Vinogradova et al., 2006) and calcium (Sanders et al., 2006) level significantly above that of ventricular cells. Calcium-stimulated adenylyl cyclases, AC1 and AC8 are known to be present in guinea pig sino-atrial node cells and our group has previously provided evidence that calcium-dependent cAMP generation contributes to the I*_f_* current measured in guinea pig sino-atrial node cells (Mattick et al., 2007).

Subjectively, conversion of cells from the linear, spontaneous, phase 4 action potential decay to the exponential rise which begets L-type calcium channel opening is slowed in cells after BAPTA exposure. This “saw-toothed” action potential shape (Figure 3B) in the presence of BAPTA is very similar to those presented as stereotyped during exposure to ryanodine in previous publications from this group which first proposed a calcium-dependence of SAN pacemaker activity (Rigg and Terrar, 1996; Rigg et al., 2000). If the “calcium clock” mechanism is dominant (Vinogradova et al., 2005) then the chelation of cytosolic calcium with a rapid and high affinity chelator such as BAPTA would be expected to effectively buffer calcium in the cleft between ryanodine receptors and the sodium-calcium exchange protein, and have effects that include suppression of local calcium release events (Bogdanov et al., 2006). Further, chelation with BAPTA may suppress other calcium-dependent events for which local calcium release events are not a requirement. For instance, the heightened diastolic calcium measured in SAN cells could itself drive a consistent inward current through NCX during all phases of the action potential (Sanders et al., 2006). Under either of these theories, chelation of cytosolic calcium would lead to a significant slowing of spontaneous diastolic depolarization before the opening of voltage-gated calcium channels by removing the depolarizing drive of NCX current.

Upon cessation of rhythmic action potential firing, we have also observed some interesting phenomena in cell membrane potential behavior. Cells which are rendered quiescent fluctuate gradually around a membrane potential in the region of −35 mV. This is very similar to the “zero-current” level previously described for the as yet unidentified background conductance of SAN myocytes (Noma and Irisawa, 1975). From this quiescent state, cells are often seen to undergo a significant membrane depolarization toward action potential threshold without actually reaching successful initiation of a complete depolarization. These events can occur as trains of distinct membrane fluctuations but are most often noted in lieu of an action potential when the cell misses one or several beats, just before firing of a sporadic action potential or burst of action potentials and during transition from any form of action potential firing back to a quiescent state. It has been shown that rapid voltage clamp of SAN myocytes is followed by several seconds of rhythmic cellular calcium transients (Vinogradova et al., 2004). The membrane potential fluctuations described here are very similar to those observed in the presence of ryanodine, which were also associated with spontaneous cellular calcium signals (Rigg et al., 2000). Whether one or a set of highly localized calcium signals are being spontaneously generated to lead to a partial depolarization by inward current through NCX, or whether these partial depolarizatioN′s themselves cause the calcium fluctuations seen by Rigg and colleagues will be interesting to discuss following future experimental work.

Data presented by Himeno et al. (2011) note that spontaneous action potential firing shows minimal interference over at least the first 30 s after patch rupture. These data have been challenged on the basis of possible changes in “seal” resistance around the patch electrode (Maltsev et al., 2011). Of particular interest in this regard is our finding that 10 mM BAPTA takes significantly longer to cause rhythm perturbations in perforated-to-whole-cell experiments than when BAPTA is applied in the absence of amphotericin/DMSO. Although it would be expected that amphotericin will not immediately compromise the SAN cell outer membrane in its entirety (it is standard practice to wait up to 15 min to achieve patch perforation for normal perforated patch recordings), the high membrane resistance of a healthy sino-atrial node cell means that the introduction of small conducting pathways can have a major influence. It cannot be ruled out that some of the changes in action potential waveforms are contributed to by this mechanism, but the similarity of these changes and the resulting action potentials to data presented during exposure of cells (Rigg et al., 2000) or tissue (Rigg and Terrar, 1996) to ryanodine supports the notion that these are attributable to a reduced contribution of calcium-dependent pathways.

In conclusion, our recordings suggest that the presence of cytosolic calcium is essential for the maintenance of normal rhythmic activity in isolated guinea pig SAN myocytes. The exact mechanism(s) which require this cytosolic calcium in order to maintain physiological function are still a matter for future investigation.

### Conflict of interest statement

The authors declare that the research was conducted in the absence of any commercial or financial relationships that could be construed as a potential conflict of interest.
